# One-Stage Total Laparoscopic Treatment for Colorectal Cancer With Synchronous Metastasis. Is It Safe and Feasible?

**DOI:** 10.3389/fsurg.2021.752135

**Published:** 2021-11-18

**Authors:** Giuseppe Sena, Arcangelo Picciariello, Fabio Marino, Marta Goglia, Aldo Rocca, Roberto L. Meniconi, Gaetano Gallo

**Affiliations:** ^1^Department of Vascular Surgery, “Pugliese-Ciaccio” Hospital, Catanzaro, Italy; ^2^Department of Emergency and Organ Transplantation, University Aldo Moro, Bari, Italy; ^3^Unit of Surgery, National Institute of Gastroenterology “Saverio de Bellis,” Research Hospital, Castellana Grotte, Italy; ^4^Department of General Surgery, “La Sapienza” University of Rome—Sant'Andrea University Hospital, Rome, Italy; ^5^Department of Medicine and Health Sciences “V. Tiberio,” University of Molise, Campobasso, Italy; ^6^Department of General Surgery and Liver Transplantation, San Camillo Forlanini Hospital, Rome, Italy; ^7^Department of Medical and Surgical Sciences, University of Catanzaro, Catanzaro, Italy

**Keywords:** colorectal cancer, liver synchronous metastasis, simultaneous laparoscopic resection, outcomes, timing, one stage treatment

## Abstract

Liver is the main target organ for colorectal cancer (CRC) metastases. It is estimated that ~25% of CRC patients have synchronous metastases at diagnosis, and about 60% of CRC patients will develop metastases during the follow up. Although several teams have performed simultaneous laparoscopic resections (SLR) of liver and colorectal lesions, the feasibility and safety of this approach is still widely debated and few studies on this topic are present in the literature. The purpose of this literature review is to understand the state of the art of SLR and to clarify the potential benefits and limitations of this approach. Several studies have shown that SLR can be performed safely and with short-term outcomes similarly to the separated procedures. Simultaneous laparoscopic colorectal and hepatic resections combine the advantages of one stage surgery with those of laparoscopic surgery. Several reports compared the short-term outcomes of one stage laparoscopic resection with open resections and showed a similar or inferior amount of blood loss, a similar or lower complication rate, and a significant reduction of hospital stay for laparoscopic surgery respect to open surgery but much longer operating times for the laparoscopic technique. Few retrospective studies compared long term outcomes of laparoscopic one stage surgery with the outcomes of open one stage surgery and did not identify any differences about disease free survival and the overall survival. In conclusion, hepatic and colorectal SLR are a safe and effective approach characterized by less intraoperative blood loss, faster recovery of intestinal function, and shorter length of postoperative hospital stay. Moreover, laparoscopic approach is associated to lower rates of surgical complications without significant differences in the long-term outcomes compared to the open surgery.

## Introduction

Colorectal cancer (CRC) represents the third most common neoplastic disease in the world with an incidence of about 1.4 million of new cases every year causing 694.000 deaths (1). The main target organ for CRC metastases is the liver (2). It is estimated that ~20–25% of CRC patients have synchronous metastases at diagnosis, and about 60% of CRC patients will develop metastases in the course of the follow up (3–6). Surgery in association with other treatments, such as neo- or adjuvant chemoradiotherapy or the recently introduced molecular targeted-therapy, represents the only potentially curative option, and allows a significant increase in the overall survival (7, 8). The timing of hepatic and colorectal surgery has been strongly debated in last years with different approaches proposed by several authors. In particular, simultaneous resections have several advantages and, as demonstrated by various reports, do not show an increased morbidity and mortality compared to delayed hepatectomies with significant economic and biological advantages. Therefore, the only contraindications to simultaneous laparoscopic resections (SLR) are complicated CRC, high ASA score and the inability to obtain a radical resection (9–13) even though, some authors recommend performing major hepatectomies only accompanied by resection of the right-sided colon and minor hepatectomies associated with rectal resections (14).

## State-of-the-Art

The management of metastatic liver CRC is multimodal and multidisciplinary and several strategies have been described so far (15, 16). In particular, Ratti et al. (15) recently investigated, in four tertiary high volume referral centers, the role of team strategy optimization in SLR demonstrating that there were no statistically significant differences between patients operated on by the same team for both colorectal and liver resections and patients operated on by the two different teams with particular colorectal or liver skill (15).

**Figure 1:**
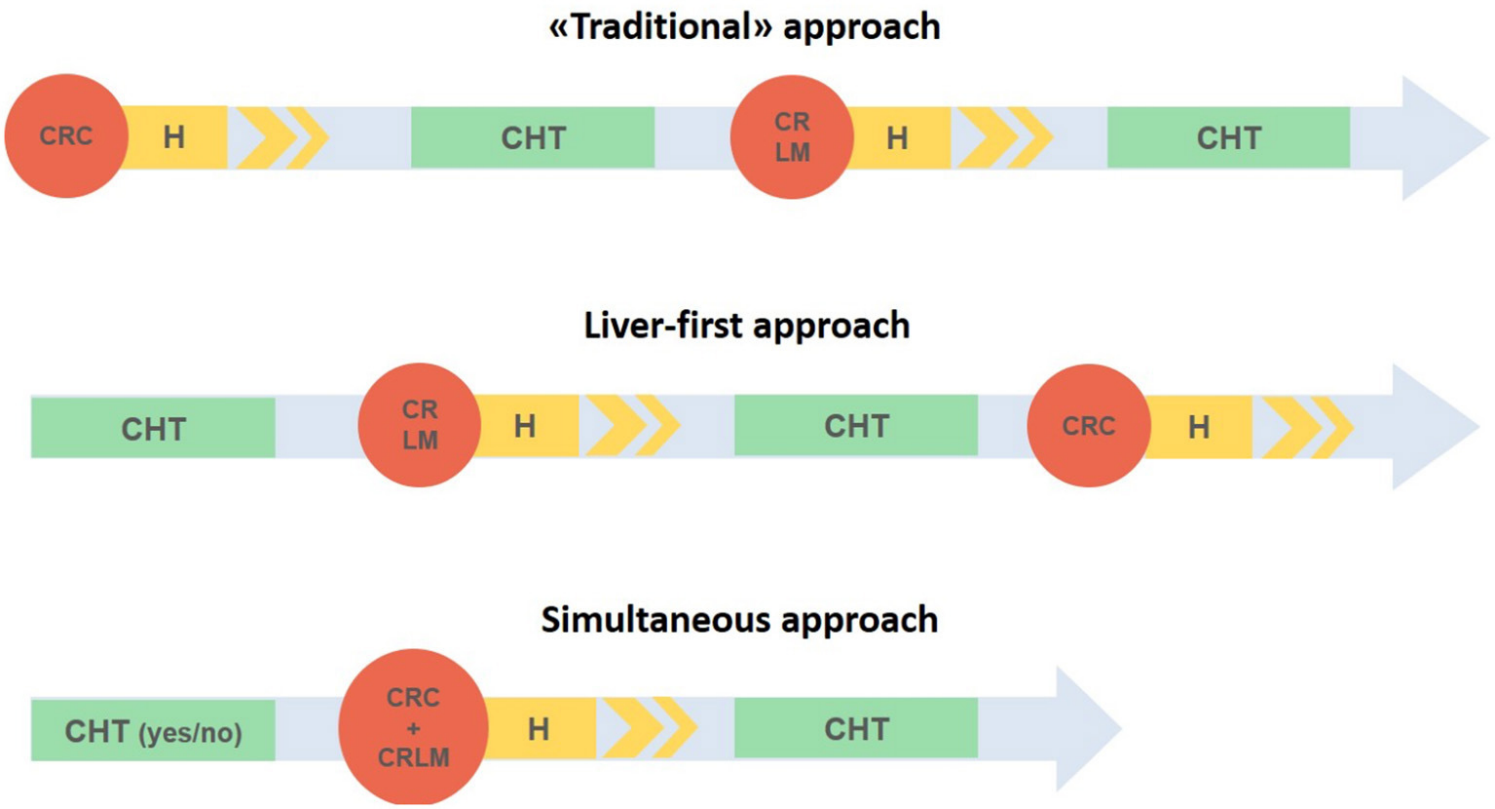
Three different strategies in case of synchronous liver metastases and colorectal cancer. CRC, Colorectal cancer; CRLM, colorectal liver metastases; H, hospital stay; CHT, chemotherapy.

Besides the SLR there are three other possibilities: the primary tumor-first approach, the liver-first approach, and the up-front hepatectomy (Figure 1).

The “traditional approach” involves the resection of the primary CRC with subsequent adjuvant therapy and then possible treatment of liver metastases after 3–6 months. While this approach reduces the risk of primary tumor progression, it exposes the patient to the possibility of unresectable liver metastases (17). Furthermore, due to complications related to colorectal resection (i.e., anastomotic leak) few patients effectively benefit from this treatment (17, 18).

The liver-first approach, the so called “chemotherapy first,” was initially described by Mentha et al. (17) and it is indicated in patients with primary asymptomatic tumors and liver metastases. It includes a preoperative chemotherapy with liver resection and a subsequent colorectal resection. In spite of the traditional approach, it is based on an immediate systemic treatment that aims to reduce the risk of progression of liver metastases as well as the possibility of downstaging the metastases which consequently might become resectable (19). In addition, it avoids unnecessary surgical treatment in chemotherapy non-responder unresectable tumors.

Lastly, the up-front hepatectomy, reported for the first time in 2008 for asymptomatic CRC and resectable liver metastases (20), includes both resections and adjuvant chemotherapy starting with the surgical treatment of liver metastases.

The introduction of minimally invasive procedures has completely transformed the surgical approach of oncological patients. Laparoscopic liver resections were introduced in the 1990s with the first publication in 1991 and 1992 (21–23), although the true spread, with major liver resections, occurred a few years later (24–26). Subsequently, the laparoscopic approach did not found great support by most surgeons due to concerns about the complexity of laparoscopically reproducing open surgery maneuvers, the difficulty of performing a satisfactory bleeding control, the risk of gas embolism and the oncological inadequacy or tumor spread risk (27, 28). Nevertheless, the technological improvements and the introduction of standardized good practices allowed the diffusion of the laparoscopic approach worldwide (29). Nowadays hepatic metastases represent one of the main indications for laparoscopy and, according to the recent Southampton Consensus Guidelines for laparoscopic liver surgery, laparoscopic liver resection has been confirmed as a valid alternative to open surgery, especially if performed by surgeons experienced in both advanced laparoscopic techniques and liver surgery (30). Recently Rocca et al. (31), in a national consensus involving 26 centers, analyzed the boundaries of minimally invasive simultaneous resections for synchronous liver metastasis and primary CRC. Although the authors produced 33 recommendations the level of evidence remains very low.

Indeed, although several teams have performed SLR of both liver and colorectal lesions, the feasibility and safety of this approach is widely debated and few studies on the subject are present in the Literature. The purpose of this review is to analyze the state of the art of SLR for synchronous liver metastases and primary CRC, identifying the potential benefits and limitations of this approach.

## Surgical Technique

**Figure 2:**
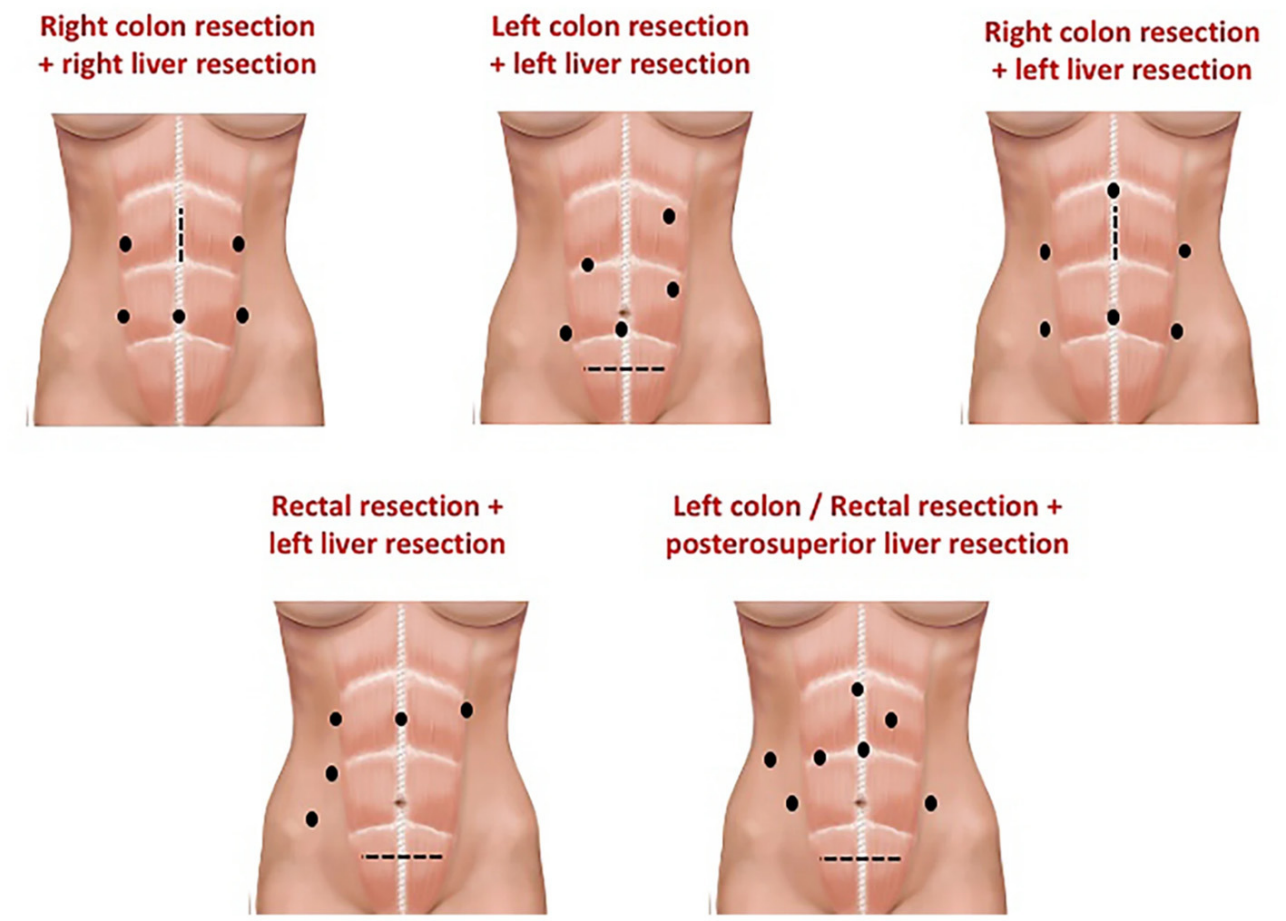
Example of trocar placement to perform a combined resection. From Rocca et al. (31).

**Figure 3:**
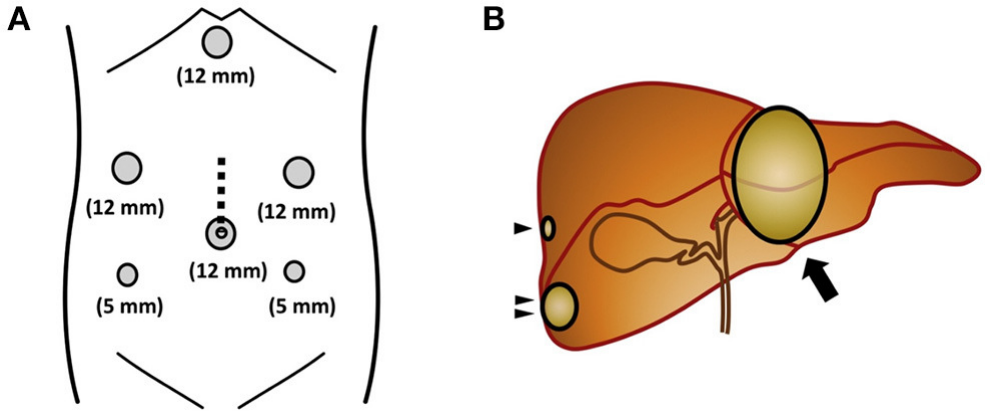
**(A)** The surgical trocar sites. The dotted line indicates the incision line used for the hand-assisted laparoscopic procedure, which was 7-cm long. **(B)** Liver scheme. Arrowhead, a tumor measuring 5 mm was found in the right hepatic lobe (segment 7); Double arrowhead, another tumor was detected in the right hepatic lobe (segment 6); Arrow, a third metastatic tumor was observed in the left hepatic lobe (segments 2/3). From Ito et al. (32).

**Figure 4:**
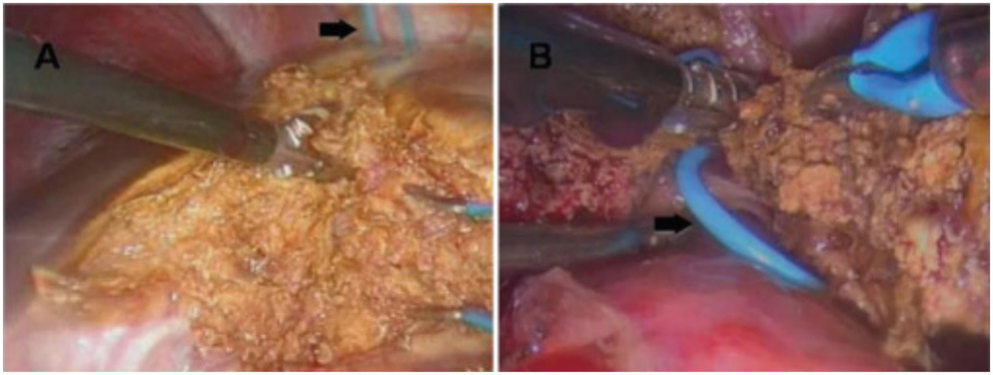
**(A)** Parenchymal transection during a left hepatectomy, performed with a thermofusion device. **(B)** The hepatic vein previously controlled with a vessel loop (black arrow) is sectioned at the end of liver division. From Tranchart et al. (34).

The placement of the trocars depends on the type of resection that will be performed (Figures 2, 3) (31, 32) and the surgical steps are performed as described by other authors (33, 34) (Figure 4).

## Advantages and Disadvantages of One Stage Laparoscopic Approach

**Figure 5:**
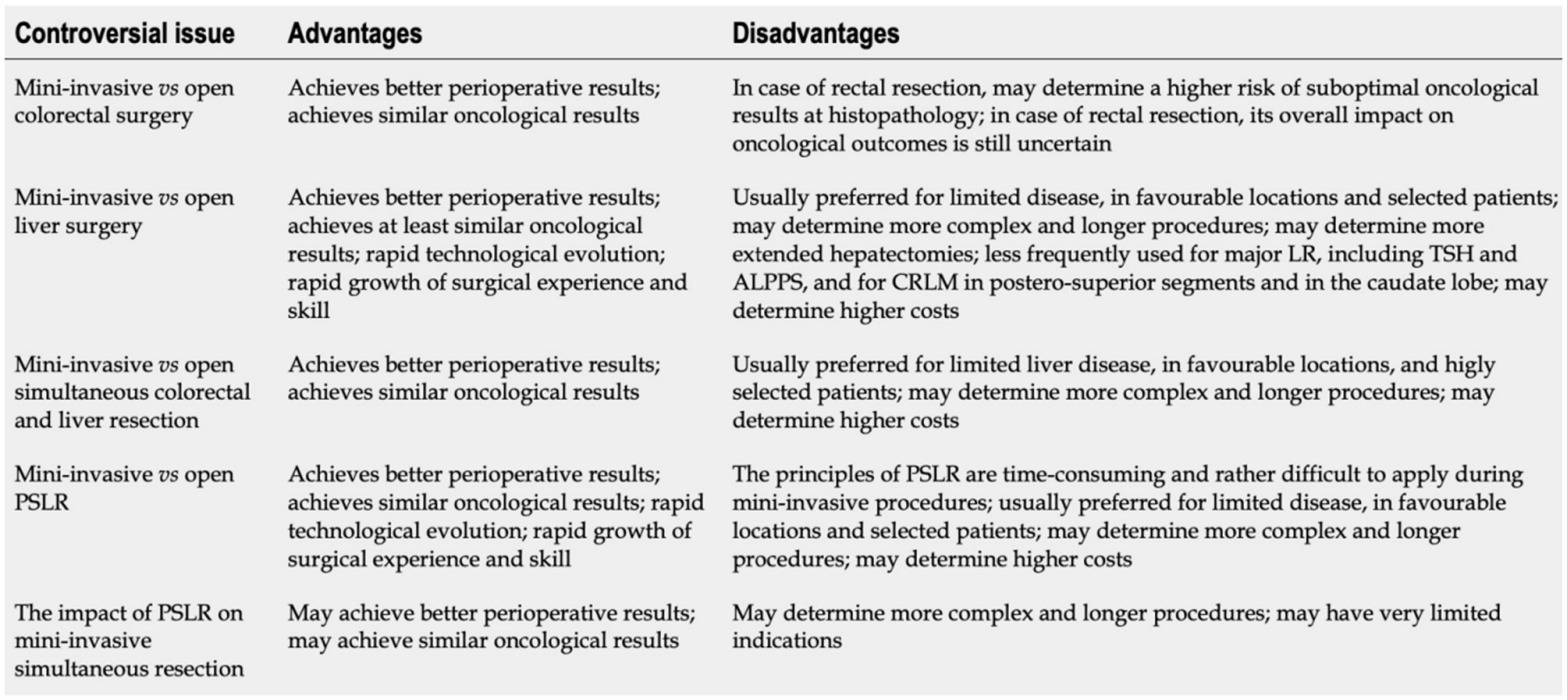
Controversial issues involving mini-invasive (laparoscopic and robotic) surgical strategies for colorectal cancer with synchronous resectable liver metastases. LR, Liver resection; TSH, Two-stage hepatectomy; ALPPS, Associating liver partition and portal vein ligation for staged hepatectomy; CRLM, Colorectal liver metastases; PSLR, Parenchymal-sparing liver resection. From De Raffele et al. (35).

SLR have several advantages and disadvantages (Figure 5) (35). The formers are represented by the execution of a single surgical procedure, the possibility of performing a complete neoadjuvant therapy, the removal of the whole macroscopic neoplastic region and the interruption of the “metastatic cascade,” and the absence of immunosuppression following the first surgery which increases metastatic cell proliferation and progression of the tumor. However, the combination of a “clean” and a “contaminated” procedure can increase the risk of septic complications (36, 37). In particular, the most frequent event is an intraoperative bacterial contamination of the liver surface.

Moreover, a technical aspect that could worsen the outcome of combined resections is the risk of anastomotic leak due to splanchnic congestion following the liver pedicle clamping (38). For this reason, Pringle maneuver should not be used routinely (28).

Usually, low rectal anastomoses present a greater risk of anastomotic leakage compared to other intestinal anastomoses (39–41).

Several studies have shown that SLR can be performed safely and with short-term outcomes similarly to the two-stage procedures (9–11, 13). Moreover, in the last few years the indications have been progressively enlarged regarding the extension of hepatic resections. Indeed, in a 19-year case series, Capussotti et al. showed that 31 patients who underwent major hepatic resections concurrent with colorectal surgery had similar mortality and morbidity rates compared to 48 patients with delayed liver surgery (3 vs. 0% and 33 vs. 33%, respectively) (12). These results were consistent with those reported by other authors (9, 13). Therefore, major hepatic resections should not be considered as absolute contraindications to SLR, but a careful patient selection is recommended. Interestingly, Ito et al. (32) demonstrated the feasibility of simultaneous resection in two elderly patients aged 78 and 83 years with ascending colon cancer and synchronous liver metastases. This study is consistent with the fact that an SLR should be considered in patients with limited liver metastases extension. Usually, in a patient with rectal cancer and a concomitant involvement of the liver that requires a major hepatectomy, it is preferred to avoid this kind of strategy (14, 42).

SLR combine the advantages of one stage surgery with the classic ones of laparoscopic surgery. An important technical advantage of laparoscopy is the magnified view which allows a better identification of the structures to be preserved (43, 44). Nevertheless, the laparoscopic approach eliminates the need for long incision laparotomy allowing less postoperative pain, faster gastrointestinal recovery and reduced bowel adhesions. Lastly, lesions located in the left anterior and lateral segments remain the best candidates for laparoscopy, even in the case of SLR. However, among the examined papers, postero-superior resections are also documented (VII and VIII segment) (45, 46).

Currently, contraindications to simultaneous resections are as follows: urgent colorectal surgery for symptomatic cancers, low performance status or high ASA score, impossibility of obtaining a radical resection. Besides these, the classic contraindications of laparoscopy such as severe heart disease, coagulation diseases, severe respiratory diseases, should be considered.

An important limitation to the laparoscopic approach of the liver is given by the need to adapt to a caudal-to-cranial view, unlike the broader vision obtained in open surgery. For this reason, lesions located very high or laterally can be difficult to be visualized (47). Moreover, laparoscopic instruments do not allow the same degree and freedom of movement as the human hand, nor the “tactile feedback.” Therefore, the mobilization of the liver is more difficult and severe bleeding cannot be controlled for a long time in laparoscopy. Despite the introduction of 3D cameras, flexible instruments, and increasingly effective and performing devices for dissection, laparoscopic liver surgery remains technically challenging and requires a long and complex learning curve. In a recent review of the Literature, evaluating 19 retrospective studies, it was shown that the learning curve was 15–64 cases for minor resections and at least 50 cases for major resections (48).

## Outcomes

**Figure 6:**
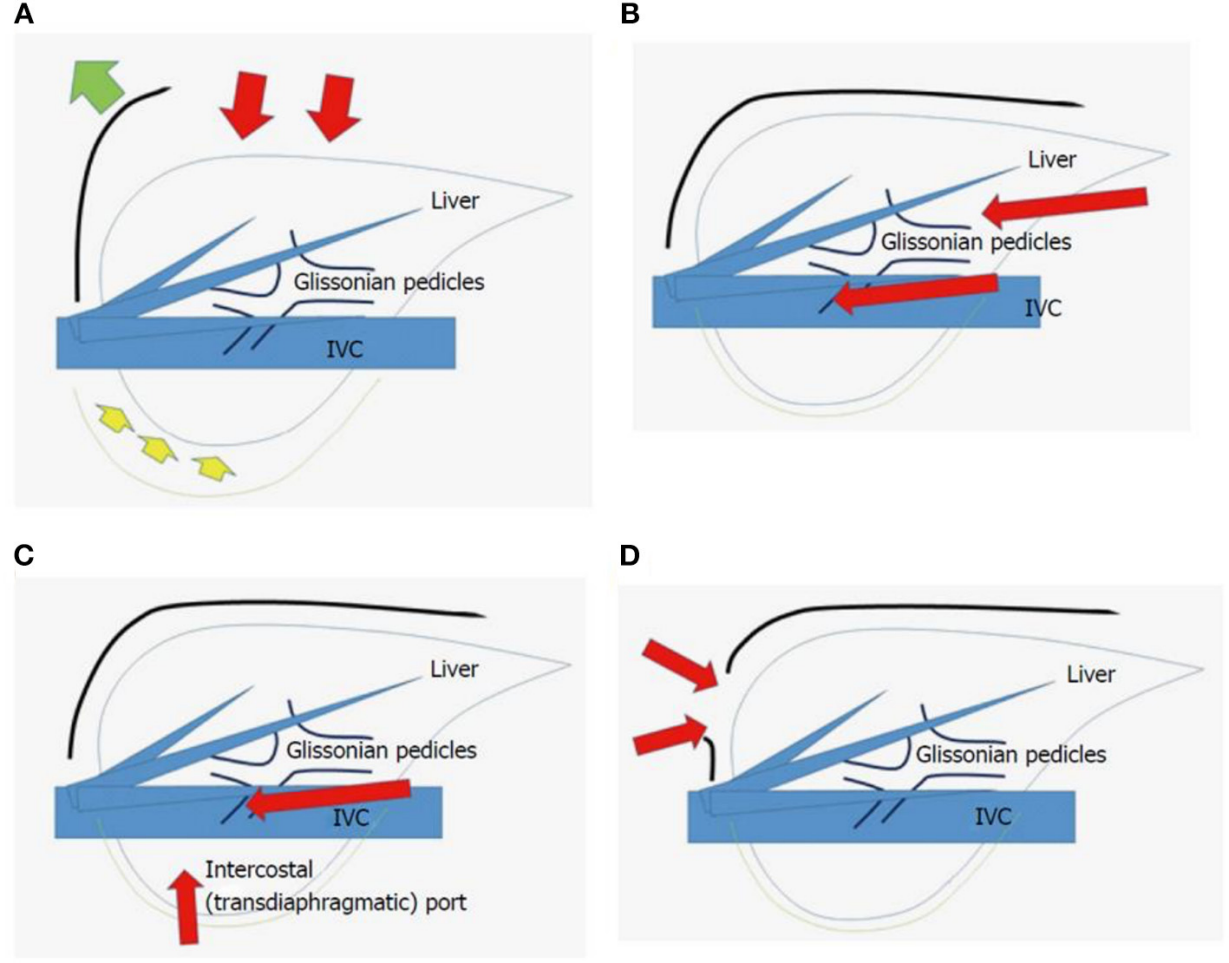
Scheme of open liver resection **(A)**, laparoscopic liver resection [regular caudal approach, **(B)**], laparoscopic liver resection [lateral approach, **(C)**] and thoracoscopic liver resection **(D)**. Red arrows indicate the directions of view and manipulation in each approach. **(A)** In the open approach, the subcostal cage containing the liver is opened with a large subcostal incision and instruments are used to lift the costal arch, after which the liver is dissected and mobilized (lifted) from the retroperitoneum; **(B)** In the regular laparoscopic caudal approach, the laparoscope and forceps are placed into the subcostal cage from the caudal direction, and the surgery is performed with minimal alteration and destruction of the associated structures; **(C)** In the laparoscopic lateral approach, the intercostal (transdiaphragmatic) ports combined with total mobilization of the liver from the retroperitoneum can allow the direct lateral approach into the cage and to the posterosuperior tumors; **(D)** Thoracoscopic approach is employed for lesions in segment 8, with direct exposure of the tumor into the pleural cavity upon incision on the diaphragm adjacent to the tumor, with the endoscope placed in the pleural cavity. From Morise and Wakabayashi (49).

There are significant differences between the open and laparoscopic approach not only from a technical point of view but also from the outcomes (Figure 6) (49).

Several reports assessed the short-term outcome of hepatic and colorectal SLR showing a similar or inferior blood loss, a similar or lower complication rate, and an important reduction of hospital stay for laparoscopic surgery respect to open surgery. On the contrary, longer operating times of laparoscopic surgery are generally reported (34, 50–56) even though in some referral centers shorter operative times are also registered (57, 58). The long-term outcomes are also comparable with the previously described cases of abdominal metastases especially at port sites (59, 60). The latter have been largely overcome thanks to some technical measures like the “no touch” technique, the specimen bag, and the abdominal wall protection (61).

The morbidity ranges between 5 and 48% for minor liver resections and between 33 and 55% for major resections (19, 20, 62).

The first studies have been published at the end of the last decade and evaluated the safety and feasibility of a simultaneous approach (63, 64).

In this context, Akiyoshi et al. showed acceptable operative time (the median total operating time was 446 min, including 222 min for colorectal resection) and blood loss (the median total estimated blood loss was 175 ml, including 10 ml for colorectal resection) with reduced complications (65).

Polignano et al. showed a shorter operating time (370 vs. 467 min, *p* = 0.005), reduced blood loss (50 vs. 40 ml, *p* = 0.02) and reduced hospital stay (7 vs.14 days; *p* = 0.1) of one stage laparoscopic surgery compared to two-stage laparoscopic surgery (66). Most of the studies considered SLR with minor hepatectomies.

After Capussotti and colleagues (12), also Tranchart et al. reported two cases of one stage major liver resections associated with colic resections in patients with large unilobular metastases, demonstrating their reliability without an increase in the complication rate (34).

Spampinato and colleagues reported a case series of 5 patients underwent major hepatectomies (67). Although with longer operating times, the results were consistent with those reported by Tranchart (34). None of the patients experienced anastomotic or bile leak and there were only 1 liver metastasis recurrences that were treated with a new laparoscopic operation.

Muangkaew et al. compared SLR, including major hepatectomies, with major liver resections alone, reporting no differences in hospital stay length (14.9 days vs. 13.3 days; *p* = 0.345), overall rate of postoperative complications (76.4 vs. 62.5 %; *p* = 0.126), colonic anastomotic leakage or sepsis, but a longer time in starting a soft diet for SLR (6.0 vs. 3.4 days; *p* < 0.001) (68).

In a recent systematic review, which examined 12 retrospective studies (4 comparative and 8 non-comparative), Moris et al. reported no differences in operating times (335.5 vs. 325.5 min) and incidence of complications between patients undergoing laparoscopic surgery and open surgery and lower blood losses for laparoscopic surgery (266.5 vs. 398 ml) (4). According to the same authors, also oncological outcomes were similar.

In a single-center and -surgeon experience considering 17 SLR, the authors reported a 94% rate of R0 resection margin on the liver and 100% distal and circumferential free-margin for the colorectal specimen (69).

Ferretti et al. (70) reported 142 laparoscopic liver resections in a SLR setting. Tumor recurrence occurred in 40 patients (28.2%) after a median follow-up of 29 (1–108) months with an overall survival of 98.8, 82.1, and 71.9% after 1-, 3, and 5-years, respectively.

From the meta-analysis by Ye et al. involving 10 cohort studies with 522 patients, it was found that minimally invasive surgery was associated with less intraoperative blood loss [weighted mean difference (WMD) = −130.09 min, *p* = 0.002] and blood transfusion (*p* = 0.03), faster recovery of intestinal function (WMD = −0.88 days, *p* = 0.01), shorter length of postoperative hospital stay (WMD = −4.06 days, *p* < 0.0001), and lower rates of surgical complications (*p* = 0.04). Interestingly, no differences were found about operating times and the rate and severity (Clavien–Dindo grade ≥ 3, *p* = 0.33) of overall complications (71). Furthermore, also the oncological outcomes OS *p* = 0.74; disease-free survival (DFS) *p* = 1.0] were also equivalent.

A more recent meta-analysis including twelve studies with 616 patients confirmed these results (72). Moreover, there has been a trend in favor of laparoscopy in terms of reduced rate of ileus, wound infection, and intra- abdominal infection. The authors concluded that SLR can be considered the first option in high-volume tertiary referral centers.

Many other retrospective studies that compared long term outcomes of laparoscopic one stage surgery with open one stage surgery did not identify differences in OS (30, 33) but only a slight difference in terms of DFS.

In the report by Shin et al., three-year OS rate of the laparoscopic group was similar to that of the open group (74.4 vs. 74.2%, *p* = 0.606). However, 3-year postoperative DFS rate of the laparoscopic group was significantly higher than that of the open group (57.8 vs. 47.4%, *p* = 0.017) (52). Consistently, Gorgun et al. reported an OS comparable between the two groups (*p* = 0.10) after a 24-month follow-up but a DFS longer in the laparoscopic group (*p* = 0.028). The two groups were comparable in terms of recurrence rates [41.3% (*n* = 12) vs. 14.2% (*n* = 2), *p* = 0.08] (54).

## Conclusion

The choice of SLR must be based on several factors such as the location, the extent and the resectability of the lesion, the general status of the patient (age, comorbidity, previous treatments) and also the experience of the surgeon.

SLR is a safe and effective approach that should be offered to patients with primary limited extension of liver metastases, characterized by less intraoperative blood loss, faster recovery of intestinal function, shorter length of postoperative hospital stay, and lower rates of surgical complications than the laparotomic approach with no significant differences in long-term outcomes. Currently, there isn't sufficient level of evidence able to demonstrate the superiority of one strategy over the others. Therefore, future reports with larger series and randomized controlled trials will be needed.

## Author Contributions

GS and GG substantial contributions to the conception and design of the work, acquisition, analysis, interpretation of data for the work, drafting the work and revising it critically for important intellectual content, final approval of the version to be published, agreement to be accountable for all aspects of the work in ensuring that questions related to the accuracy, and integrity of any part of the work are appropriately investigated and resolved. AP, FM, MG, AR, and RM substantial contributions to the conception and design of the work, acquisition, analysis, interpretation of data for the work, agreement to be accountable for all aspects of the work in ensuring that questions related to the accuracy, and integrity of any part of the work are appropriately investigated and resolved. All authors contributed to the article and approved the submitted final version.

## Conflict of Interest

The authors declare that the research was conducted in the absence of any commercial or financial relationships that could be construed as a potential conflict of interest.

## Publisher's Note

All claims expressed in this article are solely those of the authors and do not necessarily represent those of their affiliated organizations, or those of the publisher, the editors and the reviewers. Any product that may be evaluated in this article, or claim that may be made by its manufacturer, is not guaranteed or endorsed by the publisher.
